# A Note on the Prophylactic Value of Sclavo's Serum

**Published:** 1911-02-11

**Authors:** 


### Resident Medical Officers' Department.

### A NOTE ON THE PROPHYLACTIC VALUE OF SCLAVO'S SERUM.

J. B., a butcher, was admitted on January 12, 1911, with a well-marked anthrax pustule on the front of his right forearm. He stated that he was asked by a farmer on the night of December 30 last to "dress" a bullock. The farmer told him he had to kill the animal for what he thought was "obstruction."

J. B. found the abdomen greatly distended, the limbs were not stiff, the flesh was dark coloured and soft, and the spleen enlarged to three or four times the normal. The animal was evidently on the point of dying when its throat was cut. No one suspected until the veterinary surgeon saw the carcass that the condition might be "splenic fever." The sanitary inspector was then asked to see it. He recognised the gravity of the situation and immediately took the usual precautions. A pig ate some of the bullock's blood and died within twenty-four hours. The carcasses of both animals were cremated.

J. B. was not in very good health at the time. He had a very heavy week and had taken cold a day or two previously.

At 4 a.m. on the 31st he awakened feeling very sick, and vomited; during the day he improved, and the next day he felt perfectly well. He attributed his sickness to the unpleasant odour of the animal and to the fact that he was feeling more tired than usual. The sanitary inspector gave his opinion on Monday, January 2, that the animal died of splenic fever, and advised the farmer to go to the nearest hospital and receive a prophylactic dose of anti-anthrax serum.

At the inspector's suggestion J. B. accompanied the farmer and received the same dose, 20 c.c., under the skin of the abdomen.

It may be remarked that the animal was so far gone that the farmer was able to cut its throat without assistance, and only his hands were smeared with blood. The butcher, on the other hand, had his hands in the abdomen and thorax of the animal for some time, while removing the different organs. He is certain that he had no scratch about any part of his hands or arms. He was able to follow his ordinary occupation until January 8, when a small red pimple appeared on the front of his right forearm, about the junction of lower and middle thirds. He paid no particular attention to it until the following day, when he began to have pain in the right axilla.

On Tuesday the 10th the pain increased; he saw his medical attendant, who advised him to poultice the place on his forearm, which by that time had the appearance of a hard boil. On Wednesday the skin around was red and hard, and the condition looked like a blister with a brown or almost black depressed centre. The pain in his axilla was absent, although he could feel several lumps there.

On the 12th he was advised to visit the hospital; on his admission he received 30 c.c. (thirty cubic centimetres) of Sclavo's serum, the pustule was excised with half an inch of healthy skin all round—an area of two and a half inches diameter—and the wound was cauterised with pure carbolic acid.

On the following day 20 c.c. of serum were again injected. This injection was immediately followed by a rise of temperature, otherwise the temperature remained normal throughout. The patient progressed favourably without any further injections.

The wound on the forearm was allowed to heal by granulation.

